# The Impact of DDR Gene Mutations on the Efficacy of Etoposide Plus Cisplatin in Grade 3 Metastatic Gastroenteropancreatic (GEP)—Neuroendocrine Carcinoma (NEC)

**DOI:** 10.3390/cancers17152436

**Published:** 2025-07-23

**Authors:** Ji Eun Shin, Minsuk Kwon, Sung Hee Lim, Jung Yong Hong, Seung Tae Kim

**Affiliations:** Division of Hematology-Oncology, Department of Medicine, Samsung Medical Center, Sungkyunkwan University School of Medicine, Seoul 06351, Republic of Korea

**Keywords:** neuroendocrine carcinoma (NEC), DNA damage response (DDR), cisplatin-based chemotherapy, predictive biomarker

## Abstract

Purpose: Neuroendocrine carcinomas (NECs) are aggressive tumors treated with cisplatin-based chemotherapy, though responses vary. As DNA damage response (DDR) pathways influence cisplatin sensitivity, this single-center retrospective study evaluates the efficacy of first-line cisplatin in recurrent or metastatic NEC based on DDR mutation status. Materials and Methods: This study analyzed patients with grade 3 recurrent or metastatic NEC treated with first-line etoposide plus cisplatin at Samsung Medical Center between January 2019 and September 2023. All patients underwent next-generation sequencing to determine DDR mutation status, defined by pathogenic alterations in major DNA repair pathways. Clinical outcomes were assessed per RECIST v1.1. Survival analyses were conducted using Kaplan–Meier methods and Cox regression models, with significance set at *p* ≤ 0.05. Results: A total of 40 patients with NEC were included in this study. There were 16 patients with DDR wild-type (WT) and 24 patients with DDR mutant type (MT). The most common primary tumor sites were the pancreas (25.0%), stomach (20.0%), and gallbladder/duct (12.5%). Among 40 patients, those with DDR mutations (*n* = 24) showed significantly higher objective response (58.3% vs. 12.5%) and disease control rates (91.7% vs. 50.0%) compared to patients with DDR WT (*n* = 16). The median progression-free survival (PFS) showed the favorable trend in the DDR mutant group (8.0 vs. 4.3 months; *p* = 0.15), with similar trends observed across homologous recombination repair (HRR), Fanconi anemia (FA), and mismatch repair (MMR) subgroups. Conclusions: This study revealed that patients with DDR mutations had significantly higher response to first-line etoposide–cisplatin, suggesting DDR mutation status as a potential predictive marker to guide treatment and improve outcomes in recurrent or metastatic NEC.

## Simple Summary

Neuroendocrine carcinoma (NEC) is an aggressive malignancy with limited treatment options and variable response to standard platinum-based chemotherapy. Given the role of DNA damage response (DDR) pathways in mediating sensitivity to cisplatin, this study investigated whether DDR gene alterations could serve as predictive biomarkers for response to first-line etoposide–cisplatin in patients with recurrent or metastatic NEC. By analyzing clinical outcomes according to DDR mutation status, this study found that patients harboring DDR mutations experienced significantly higher response and disease control rates. These findings suggest that DDR mutation profiling may guide therapeutic decisions and improve clinical outcomes in this challenging disease.

## 1. Introduction

Neuroendocrine carcinomas (NECs) represent a heterogeneous group of aggressive malignancies characterized by neuroendocrine differentiation, exhibiting both morphological and functional attributes of neurosecretory cells [1]. These neoplasms can arise across diverse anatomical sites, including the gastroenteropancreatic (GEP) tract [2,3]. Due to their intrinsic propensity for rapid proliferation and early dissemination, the management of advanced stage NECs necessitates effective systemic therapeutic interventions [4].

A fundamental chemotherapeutic regimen for extrapulmonary poorly differentiated NECs involves the cytotoxic combination of etoposide and cisplatin [1]. While this platinum-based doublet can induce initial tumor regression in a significant proportion of patients, the clinical outcomes exhibit considerable variability between patient subsets, underscoring the critical need to find determinants of therapeutic response [5].

DNA damage response (DDR) constitutes a complex and interconnected network of cellular pathways essential for preserving genomic stability. These pathways detect diverse DNA lesions, trigger signaling cascades, and orchestrate the repair of damaged DNA. Disruption of these mechanisms can result in the accumulation of mutations and genomic instability, which are known as key characteristics of cancer. Major DDR pathways include non-homologous end joining (NHEJ), mismatch repair (MMR), nucleotide excision repair (NER), homologous recombination (HR), and the Fanconi anemia (FA) pathway [6].

Chemotherapeutic agents such as cisplatin exert their antineoplastic effects by inducing genotoxic stress, primarily through the formation of DNA adducts, including intra-strand and inter-strand crosslinks [7]. The therapeutic success of cisplatin is basically predicated upon the balance between the extent of cytotoxic DNA lesions induced within the neoplastic cells and their inherent DNA repair proficiency mediated by robust DDR pathways [8].

Consequently, the functional status of the DDR machinery within neoplastic cells is a critical determinant of treatment sensitivity and the potential for acquired chemoresistance [9]. Accumulating evidence across various cancer reveals that somatic and germline alterations in DDR genes can serve as predictive biomarkers for the therapeutic efficacy of cisplatin-based chemotherapy [8,10,11,12,13,14,15,16].

This study is a single-center observational study aimed at evaluating the efficacy of first-line cisplatin treatment according to DDR mutation status in patients with recurrent or metastatic NEC.

## 2. Materials and Methods

### 2.1. Study Design and Participants

We conducted an analysis on patients diagnosed with grade 3 recurrent/metastatic NEC at the Samsung Medical Center from January 2019 to September 2023. All patients had histologically confirmed NEC; received etoposide plus cisplatin as a first-line therapy (via a regimen consisting of cisplatin 60 mg/m^2^ administered intravenously on day 1 and etoposide 100 mg/m^2^ administered intravenously on days 1 to 3, repeated every 3 weeks); and simultaneously underwent next-generation sequencing (NGS) via tissue of malignancy or blood.

We reviewed the electronic clinical records and extracted data on age, sex, Eastern Cooperative Oncology Group (ECOG) performance status, primary site, histopathology, DDR mutation status, and distant metastasis at initiation of treatment.

DDR mutation status was identified using NGS with the TruSight Oncology 500 assay (Illumina, San Diego, CA, USA) and Oncomine Focus assay (ThermoFisher, Waltham, MA, USA). For this study, DDR mutation was defined as the presence of one or more alterations in genes involved in major DNA repair pathways; homologous recombination repair (HRR; BRCA1, BRCA2, ATM, BARD1, BRIP1, CHEK2, PALB2, RAD51, RAD51C, RAD51D, RAD54L), non-homologous end joining (NHEJ; Ku70 (XRCC6), Ku80 (XRCC5), XRCC4, DNA-PKcs (PRKDC), LIG4), nucleotide excision repair (NER; ERCC1, ERCC2 (XPD), ERCC3 (XPB), ERCC4 (XPF), ERCC5 (XPG), ERCC6, ERCC8), base excision repair (BER; OGG1, APEX1, XRCC1, POLB, LIG3), mismatch repair (MMR; MLH1, MSH2, MSH6, PMS2), Fanconi anemia (FA; FANCA, FANCB, FANCC, FANCD1 (BRCA2), FANCD2, FANCE, FANCF, FANCG, FANCI, FANCJ (BRIP1), FANCL, FANCM, FANCN) pathway, and other DDR-related genes such as CHEK1 and CHEK2. TP53 was excluded. Patients were classified as DDR mutant type (DDR MT) if they harbored at least one pathogenic or likely pathogenic mutation in any of the aforementioned DDR pathway genes; otherwise, they were categorized as DDR wild-type (DDR WT).

### 2.2. Outcomes

Clinical outcomes were evaluated for objective response rate (ORR), disease control rate (DCR), progression-free survival (PFS), and overall survival (OS). Tumor response was evaluated as complete response (CR), partial response (PR), stable disease (SD), or progressive disease (PD), according to the Response Evaluation Criteria in Solid Tumor, version 1.1. ORR was defined as the percentage (%) of patients with confirmed CR or PR. PFS was measured from the start of the treatment to the date of disease progression or death from any cause using RECIST 1.1. OS was calculated from the start of the treatment to the date of death from any cause.

### 2.3. Statistical Analysis

Categorical and continuous variables were summarized using descriptive statistics. Survival analyses were performed using Kaplan–Meier curves and compared using log-rank tests. Cox proportional hazards regression models were used to obtain estimates of hazard ratios (HRs) based on multivariate analysis. All *p* values were two-sided, and confidence intervals (CIs) were at the 95% level, with statistical significance defined as *p* ≤ 0.05. All statistical analyses were performed using IBM SPSS Statistics 27 and R version 4.4.3. The data cutoff date was 28 February 2025.

## 3. Results

### 3.1. Clinical Characteristics

Forty patients with NEC were included in this study. There were 16 patients DDR WT and 24 patients with DDR MT. Baseline characteristics are summarized in Table 1.

The median age was 64.7 years (range: 45–78). Males comprised 65.0% of the total population, and most patients had good performance status (PS), with 97.5% having an FULL MAME (ECOG) PS of 0 or 1. The most common primary tumor site was followed by the pancreas (25.0%), stomach (20.0%), and gallbladder/duct (12.5%). Other sites included esophagus (2.5%), small bowel (2.5%), rectum (10.0%), and unknown or other locations (27.5%). Liver metastases were the most common site of distant metastasis, occurring in 65.0% of patients, followed by bone metastases (7.5%), peritoneal metastases (10.0%), and lymph node involvement in 55.0%. The clinical characteristics mentioned above were well balanced between the DDR WT and DDR MT groups, showing no significant differences.

### 3.2. Distribution of DDR Pathway Mutations

Among the 24 patients classified as DDR mutant (DDR MT), mutations were most frequently observed in the homologous recombination repair (HRR) pathway, affecting 66.7% of patients. The most commonly mutated genes within HRR were BRCA1 and BRCA2, each identified in nine patients, followed by PALB2 (three patients), ATM four patients), and RAD52 (one patient). Defects in the mismatch repair (MMR) pathway were less common, observed in 16.7% of patients, with mutations primarily in MLH1 (three patients) and MSH6 (one patient). Mutations in the Fanconi anemia (FA) pathway were present in 33.3% of patients, predominantly involving FANCA, FANCI, and FANCG. Among patients with HRR, MMR, and FA mutations, three had concurrent mutations in both HRR and MMR pathways; one had mutations in both HRR and FA; and one had mutations in both MMR and FA. No mutations were detected in genes associated with non-homologous end joining (NHEJ), nucleotide excision repair (NER), or base excision repair (BER) pathways (Table 2).

### 3.3. Efficacy of Cisplatin According to DDR Mutations

#### 3.3.1. Objective Response Rate (ORR)

Among the 40 patients treated with cisplatin-based chemotherapy, response rates differed significantly between the DDR WT and DDR MT groups (Table 3). Progressive disease (PD) was observed in 25.0% of DDR WT patients compared to only 4.2% of DDR MT patients, indicating better disease control in the DDR MT group. Also, complete response (CR) was observed in none of the DDR WT patients and 8.3% of DDR MT patients, while partial response (PR) was seen in 12.5% of DDR WT patients and 50.0% of DDR MT patients. Stable disease (SD) occurred in 37.5% of DDR WT patients and 33.3% of DDR MT patients.

The objective response rate (ORR), defined as the proportion of patients achieving PR or CR, was significantly higher in the DDR MT group at 58.3%, compared to 12.5% in the DDR WT group. Similarly, the disease control rate (DCR)—which includes PR, CR, and SD—was markedly higher in the DDR MT group at 91.7%, compared to 50.0% in the DDR WT group.

#### 3.3.2. Progression-Free Survival (PFS)

**Figure 1:**
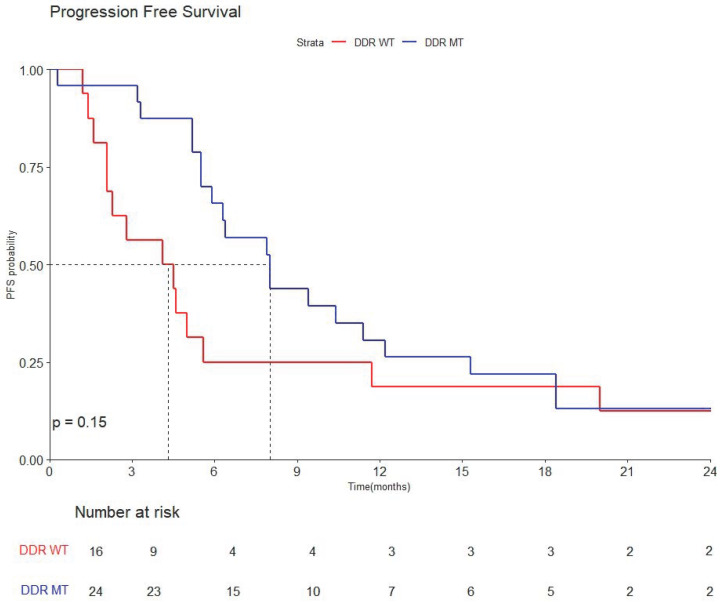
Kaplan–Meier curve of progression-free survival by DDR mutations.

The median follow-up duration for the total population was 36.3 months (range: 25.0–63.5). The median progression-free survival (PFS) for all patients was 5.9 months (95% CI, 5.2–10.4). Patients with DDR mutations demonstrated a longer median PFS of 8.0 months (95% CI, 5.9–15.3) compared to 4.3 months (95% CI, 2.1–20.0) in the DDR WT group (HR 0.61; 95% CI, 0.31–1.2) (*p* = 0.15) (Figure 1).

In particular, regarding progression-free survival (PFS) based on BRCA1/2 mutation status, a total of 11 patients had BRCA mutations. The median PFS was slightly longer in patients with BRCA mutations (6.4 months; 95% CI, 5.2–NA) compared to those without (5.6 months; 95% CI, 4.6–12.2), although the difference was not statistically significant (HR 0.9; 95% CI, 0.48–2.3; *p* = 0.93).

**Figure 2:**
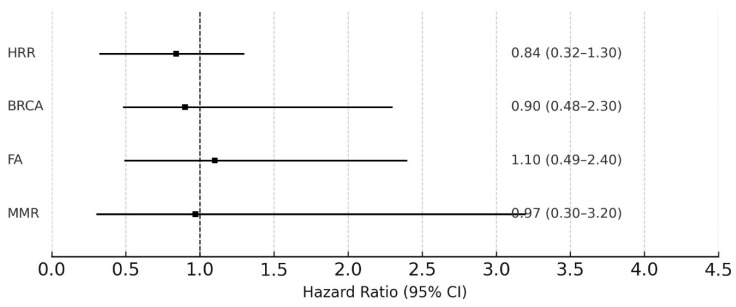
Progression-free survival according to DDR pathway mutations.

In terms of other DDR pathways, patients with mutations in HRR, FA, and MMR genes showed numerically longer or similar PFS compared to wild-type patients—HRR: 8.0 months (95% CI, 5.9–NA) vs. 5.0 months (95% CI, 2.8–11.7); FA: 7.95 months (95% CI, 5.5–NA) vs. 5.6 months (95% CI, 4.6–11.7); MMR: 5.7 months (95% CI, 0.3–NA) vs. 6.3 months (95% CI, 5.0–11.4). However, these differences were not statistically significant (HRs not significant; *p* = 0.21, 0.87, and 0.97, respectively) (Figure 2).

#### 3.3.3. Overall Survival (OS)

**Figure 3:**
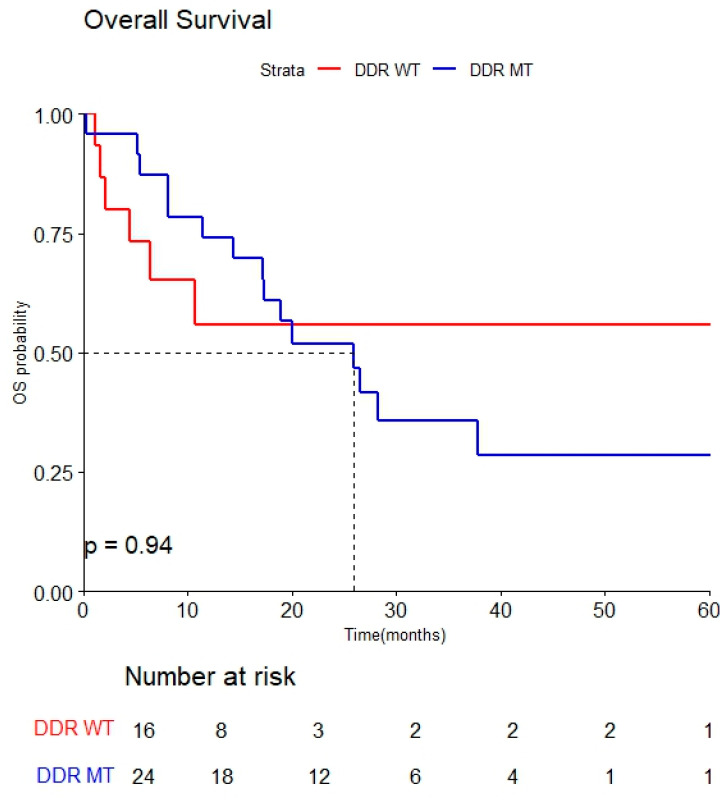
Kaplan–Meier curve of overall survival by DDR mutations.

The median overall survival (OS) was 26.0 months (95% CI, 17.2–NA). When stratified by DDR mutation status, patients with DDR mutations had a median OS of 26.0 months (95% CI, 17.2–NA), while the median OS for DDR wild-type patients was not reached (lower 95% CI, 6.4) group (HR 0.58; 95% CI, 0.29–1.2) (Figure 3).

## 4. Discussion

In this study, the clinical outcomes of patients with recurrent/metastatic NEC who received first-line etoposide–cisplatin were analyzed according to their DDR mutation status. Among the 40 patients, 24 had DDR mutations. The objective response rate was significantly higher in the mutation (MT) group compared to the wild-type (WT) group (ORR: 58.3% vs. 12.5%; DCR: 91.7% vs. 50.0%). Progression-free survival (PFS) was also superior in the DDR MT group (8.0 months vs. 4.3 months; HR 0.61; 95% CI, 0.31–1.2). Subgroup analysis by pathway showed that patients with mutations in HRR, BRCA, and FA pathways had improved PFS, while MMR mutation status did not significantly affect outcomes. However, these results should be interpreted with caution, because most mutations occurred in the HRR pathway, and the number of patients with other pathway mutations was too small for reliable comparison.

DDR gene mutations enhance cisplatin efficacy by impairing cancer cells’ ability to repair cisplatin-induced DNA lesions, leading to accumulated genomic damage and apoptosis. Cisplatin creates DNA crosslinks and adducts that activate DDR pathways to initiate repair or trigger cell death [17]. However, mutations in DDR genes disrupt critical repair mechanisms, leaving cisplatin-induced DNA damage unresolved [8]. For example, ERCC2 mutations reduce the helicase activity required for NER, while BRCA1/2 defects prevent HR-mediated repair of double-strand breaks [18]. This genomic instability overwhelms cellular survival pathways, increasing cisplatin-induced cytotoxicity. These mutations also correlate with enhanced sensitivity to combination therapies, as unresolved DNA damage synergizes with radiation or PARP inhibitors [19,20].

The correlation between DDR gene mutations and cisplatin efficacy has already been demonstrated in various cancer types in clinical settings. In metastatic castration-resistant prostate cancer (mCRPC), patients with DDR gene alterations showed better responses to platinum chemotherapy [10]. Similarly, in metastatic urothelial carcinoma, DDR alterations were associated with improved PFS and OS in patients treated with platinum-based chemotherapy [8,15]. This was further supported by findings in advanced non-small cell lung cancer (NSCLC) where DDR gene mutations were linked to better PFS and OS with platinum-containing regimens [12]. Conversely, in head and neck squamous cell carcinoma (HNSCC), lower nucleotide excision repair (NER) capacity, influenced by DDR genes, correlated with longer PFS in patients receiving cisplatin-based therapy [21]. Moreover, in digestive high-grade neuroendocrine neoplasms (HG-NEN), specific DDR gene mutations like TP53 and BRAF have shown predictive value for response to cisplatin-based chemotherapy [22]. These findings suggest that DDR status can serve as a potential biomarker for predicting patient selection and response to cisplatin therapy in multiple malignancies.

In this study, although the results were not statistically significant, they were consistent with previous research findings. However, regarding OS, the median value for DDR WT was not reached, whereas the median was 26.0 months in the DDR MT group. In the DDR WT group, two patients have been surviving for over 50 months without any OS events, and both maintained disease stability after receiving local therapy (radiation) following chemotherapy, which is believed to have influenced the results.

This study has several limitations. The retrospective, single-center design and small sample size (N = 40) reduced statistical power and generalizability. Most DDR mutations clustered in the HRR pathway, limiting meaningful subgroup comparisons across other pathways. Incomplete data on post-treatment interventions, including second-line therapies and radiotherapy, restricted our ability to evaluate their impact on overall survival. Additionally, because the cohort was small with few outcome events, multivariate analysis to adjust for confounders such as pancreatic primary could not be performed. Finally, we did not analyze outcomes by pathological subtype due to small numbers and variability in histologic classification. These limitations highlight the need for validation in larger, prospective studies.

Nevertheless, this study revealed the clinical relevance of DDR mutation status in predicting the efficacy of first-line etoposide–cisplatin in patients with recurrent or metastatic NEC. These findings support the potential utility of DDR mutation profiling in guiding treatment strategies and optimizing clinical outcomes in NEC.

## 5. Conclusions

This study suggests that DDR gene mutations may serve as a predictive biomarker for improved response to first-line etoposide–cisplatin in patients with recurrent or metastatic grade 3 GEP-NEC, demonstrating higher response rates and longer PFS in patients with DDR mutations. These results support the clinical relevance of DDR profiling in guiding treatment decisions.

## Figures and Tables

**Table 1:** Baseline characteristics.

Patient Characteristics	Total (*n* = 40)	DDR WT (*n* = 16)	DDR MT (*n* = 24)	*p*-Value
Age (years), median (range)	64.7 ± 9.2	66.2 ± 11.0	63.7 ± 7.9	0.418
Sex				0.543
Male	26 (65.0%)	9 (56.2%)	17 (70.8%)	
Female	14 (35.0%)	7 (43.8%)	7 (29.2%)	
ECOG performance status				0.686
0	2 (5.0%)	1 (6.2%)	1 (4.2%)	
1	37 (92.5%)	15 (93.8%)	22 (91.7%)	
2	1 (2.5%)	0 (0.0%)	1 (4.2%)	
Primary site				0.225
Esophagus	1 (2.5%)	1 (6.2%)	0 (0.0%)	
Stomach	8 (20.0%)	3 (18.8%)	5 (20.8%)	
Pancreas	10 (25.0%)	7 (43.8%)	3 (12.5%)	
Small bowel	1 (2.5%)	0 (0.0%)	1 (4.2%)	
GB/Duct	5 (12.5%)	2 (12.5%)	3 (12.5%)	
Rectum	4 (10.0%)	0 (0.0%)	4 (16.7%)	
Others	3 (7.5%)	1 (6.2%)	2 (8.3%)	
Unknown	8 (20.0%)	2 (12.5%)	6 (25.0%)	
Pathology				0.618
SCNEC	5 (12.5%)	1 (6.2%)	4 (16.7%)	
LCNEC	1 (2.5%)	0 (0.0%)	1 (4.2%)	
Other G3 NEC	22 (55.0%)	10 (62.5%)	12 (50.0%)	
NEC NOS	12 (30.0%)	5 (31.2%)	7 (29.2%)	
Ki-67				0.856
20–55%	11 (27.5%)	5 (31.3%)	6 (25.0%)	
≥55%	17 (42.5%)	6 (37.5%)	11 (45.8%)	
Unknown	12 (30.0%)	5 (31.3%)	7 (29.2%)	
Distant metastatic lesions				
Liver	26 (65.0%)	13 (81.2%)	13 (54.2%)	0.155
Lung	0 (0.0%)	0 (0.0%)	0 (0.0%)	
Bone	3 (7.5%)	0 (0.0%)	3 (12.5%)	0.391
Peritoneum	4 (10.0%)	2 (12.5%)	2 (8.3%)	1.000
LN	22 (55.0%)	9 (56.2%)	13 (54.2%)	1.000

ECOG, Eastern Cooperative Oncology Group; GB, gallbladder; SCNEC, small cell neuroendocrine carcinoma; LCNEC, large cell neuroendocrine carcinoma; LN, lymph nodes.

**Table 2:** Distribution of DDR gene mutations.

DDR Pathway	Patients with Mutation (*n* = 24)	Mutated Genes (Frequency)
HRR	16 (66.7%)	BRCA1 (9), BRCA2 (9), PALB2 (3), ATM (4), ATR (1), RAD52 (1)
MMR	4 (16.7%)	MLH1 (3), MSH6 (1)
FA	8 (33.3%)	FANCA (3), FANCI (3), FANCG (3), FANCC (1), FANCF (1)
NEHJ/NER/BER	0	

HRR, homologous recombination repair; MMR, mismatch repair; FA, Fanconi anemia; NEHJ, non-homologous end joining; NER, nucleotide excision repair; BER, base excision repair.

**Table 3:** Objective response rates stratified by DDR gene mutations.

Treatment Response	DDR WT (*n* = 16)	DDR MT (*n* = 24)
Overall ORR, No. (%)		
Complete response	0 (0.0%)	2 (8.3%)
Partial response	2 (12.5%)	12 (50.0%)
Stable disease	6 (37.5%)	8 (33.3%)
Progression disease	4 (25.0%)	1 (4.2%)
Not evaluated	4 (25.0%)	1 (4.2%)
Objective response rate, %	12.5%	58.3%
Disease control rate, %	50.0%	91.7%

## Data Availability

The original contributions presented in this study are included in the article. Further inquiries can be directed to the corresponding author.
